# Design of Promising Green Cation-Exchange-Membranes-Based Sulfonated PVA and Doped with Nano Sulfated Zirconia for Direct Borohydride Fuel Cells

**DOI:** 10.3390/polym13234205

**Published:** 2021-11-30

**Authors:** Marwa H. Gouda, Noha A. Elessawy, Sami A. Al-Hussain, Arafat Toghan

**Affiliations:** 1 Polymer Materials Research Department, Advanced Technology and New Materials Research Institute (ATNMRI), City of Scientific Research and Technological Applications City (SRTA-City), Alexandria 21934, Egypt; marwagouda777@yahoo.com; 2 Computer Based Engineering Applications Department, Informatics Research Institute IRI, City of Scientific Research and Technological Applications City (SRTA-City), Alexandria 21934, Egypt; nony_essawy@yahoo.com; 3 Chemistry Department, College of Science, Imam Mohammad Ibn Saud Islamic University (IMSIU), Riyadh 11623, Saudi Arabia; sahussain@imamu.edu.sa; 4 Chemistry Department, Faculty of Science, South Valley University, Qena 83523, Egypt

**Keywords:** direct borohydride fuel cell, cation exchange membrane, poly(vinyl alcohol), iota carrageenan, sulfated zirconia

## Abstract

The direct borohydride fuel cell (DBFC) is a low-temperature fuel cell that requires the development of affordable price and efficient proton exchange membranes for commercial purposes. In this context, super-acidic sulfated zirconia (SO_4_ZrO_2_) was embedded into a cheap and environmentally friendly binary polymer blend, developed from poly(vinyl alcohol) (PVA) and iota carrageenan (IC). The percentage of SO_4_ZrO_2_ ranged between 1 and 7.5 wt.% in the polymeric matrix. The study findings revealed that the composite membranes’ physicochemical features improved by adding increasing amounts of SO_4_ZrO_2_. In addition, there was a decrease in the permeability and swelling ratio of the borohydride membranes as the SO_4_ZrO_2_ weight% increased. Interestingly, the power density increased to 76 mW cm^−2^ at 150 mA cm^−2^, with 7.5 wt.% SO_4_ZrO_2,_ which is very close to that of Nafion117 (91 mW cm^−2^). This apparent selectivity, combined with the low cost of the eco-friendly fabricated membranes, points out that DBFC has promising future applications.

## 1. Introduction

One of the most important current global challenges is finding alternative solutions to conventional energy sources such as petroleum [1]. Recently, fuel cells (FCs) have been considered as sustainable energy sources, making them an attractive and alternative category to finite reserves [2,3,4]. They can directly convert chemical energy into electrical energy [5,6,7,8,9]

One of the different types of FCs that have been developed so far is the direct borohydride fuel cell (DBFC). It has a high-power density (HPD) at relatively low operating temperatures, which makes it a promising power system for portable applications [10,11]. In addition, it is fueled by non-explosive and non-toxic reactants, which enable it to be applied in the portable and transport sectors. The DBFC provides electrical power by reducing gaseous or liquid oxidants and oxidizing borohydride ions (${\mathrm{BH}}_{4}^{-}$). Sodium borohydride (NaBH_4_) is utilized as anon-hydrocarbon liquid fuel; hence, there is no harmful emission of carbon dioxide compared to FCs fed with alcohol. In comparison to oxygen, liquid hydrogen peroxide (H_2_O_2_) is preferred as an oxidant due to its faster reduction kinetics, which generate a higher power density. This broadens the application of DBFCs in oxygen-free environments, such as in space and in submarines [12,13,14].

A membrane is used as a separator in the fuel cell, between the anode and the cathode. It plays a vital role in ion transport to maintain the balance of charges in the cell. An anion-exchange membrane (AEM) easily transfers OH^−^ from cathode to anode. However, a cation-exchange membrane (CEM) is preferably used to separate the compartments of the cathode and anode in FCs. This is mainly because CEMs can better decrease the borohydride crossover compared to AEMs, due to the electrostatic repulsion that occurs between the negative charges of ${\mathrm{BH}}_{4}^{-}$ and the CEM [12]. In addition, CEMs help to transport Na^+^ ions from the anode to the cathode. The Nafion family is widely used as perfluorinated CEMs in DBFCs [14,15,16], because it provides good chemical and mechanical stability, in addition to good ionic conductivity [12,14,15]. However, its membranes are very expensive and complicated in the manufacturing process, which limits its commercialization in fuel cells [17,18]. Due to this, it is very important to replace it with cheap, green polymeric membranes [12,14].

Non-perfluorinated polymers, such as poly(ether ether ketone) (PEEK), poly(benzimidazole) (PBI), poly(arylene ether sulfone) (PSU), and poly(styrene) (PS), are among the polymers most often used as alternatives [18,19,20,21]. However, synthesizing these non-degradable polymers takes time, generates harmful organic solvents, and uses high temperatures. These make the membrane synthesis complicated, costly, and not environmentally friendly. Therefore, researchers are looking for alternatives with green, efficient, and inexpensive polymeric films. Recently, the use of cheap, green polymers that can be biodegraded, such as polyvinyl alcohol (PVA) and iota carrageenan (IC), has become more attractive [18,22,23,24,25].

Thus, PVA is a well-suited polymer because it is chemically stable, can form film adhesion, and is hydrophilic [18,26,27]; however, its rigid, semi-crystalline structure gives it low proton conductivity. Due to this, it is seldom used as a proton-exchange membrane in FCs. Therefore, it is necessary to look for the possibility of repairing this defect. It has been found that adding dopants, or combining them with another polymer electrolyte, accomplishes this purpose [18,23,26]. For instance, PVA blended with functionalized titania [28], or sulfated/phosphated titania [29,30], enhances the membranes’ ionic conductivity due to the formation of more hydrogen bonds. Furthermore, sulfonated graphene oxide was used as a doping agent in the same polymer matrix and achieved a power density of about 65 mW·cm^−2^ [31]. PVA blended with IC is favored due to hydrogen bond interactions between the –OH groups of IC and PVA [32,33]. Moreover, IC is a commonly used biopolymer in synthesizing polymer electrolyte membranes [34] due to its non-toxic properties, chemical stability, and flexibility.

To improve the oxidative stability and the mechanical, thermal, and dimensional characteristics of the membrane, as well as its ability to hinder ${\mathrm{BH}}_{4}^{-}$ crossover and conduct ions, many researchers have commonly used a strategy such as inserting dopants into polymer structures for the production of a nanocomposite membrane [17,18,35,36]. Incorporation of sulfated zirconia (SO_4_ZrO_2_) into a polymer matrix is particularly beneficial in fuel cell applications because it is chemically stable, has a large surface area, is mechanically strong, and prevents fuel crossover [37,38,39,40,41,42,43]. SO_4_ZrO_2_ consists of hydrophilic functional groups that contain oxygen, such as sulfate groups. They can enhance water adsorption, thereby creating pathways for conducting protons [17,19,20]. When SO_4_ZrO_2_ is inserted into a polymer matrix, hydrogen bonds are formed between the oxygenated groups and –OH groups of the polymer chains in SO_4_ZrO_2_. These hydrogen bonds compress and strengthen the membrane matrix, preventing excessive water absorption and swelling [26,44,45]. In addition, the nanocomposite has more ability to conduct ions. This is as a result of the increased number of proton delivery sites, due to the presence of sulfate radicals in the structure of the nanocomposite.

In this respect, the current work aims to fabricate and develop a new SPVA/IC/SO_4_ZrO_2_ membrane that fulfills the above requirements, to take a step forward towards commercializing DBFCs. To achieve this, SO_4_ZrO_2_ was first synthesized and then added as a dopant into the matrix of SPVA/IC polymers at various concentrations. It led to the formation of a novel nanocomposite membrane called S-PVA/IC/SO_4_ZrO_2_. The oxygen groups of SO_4_ZrO_2_, including sulfate groups, are connected to the –OH groups of IC and PVA by generating hydrogen bonds, which are expected to improve the ability of the membrane to stabilize oxidation, conduct Na^+^, improve mechanical resistance, and hinder ${\mathrm{BH}}_{4}^{-}$ crossover while reducing the absorption of excess water. The performance of the DBFC can be improved with the use of such a membrane.

## 2. Materials and Methods

### 2.1. Synthesis

#### 2.1.1. Synthesis of Nano Sulfated Zirconia (SO_4_ZrO_2_)

SO_4_ZrO_2_ nanoparticles were prepared by simple calcination [46] of ammonium sulfate (NH_4_)_2_SO_4_ and zirconium oxychloride octahydrate ZrOCl_2_ 8H_2_O, with a 6:1 molar ratio, without any solvent. The powder mixture was calcined at 600 °C for 5 h, and then ground in ball mill.

#### 2.1.2. Preparation of SPVA/IC/SO_4_ZrO_2_ Membranes

Ten grams of PVA (99% hydrolysis and medium mW, USA) was dissolved in 100 mL of deionized H_2_O at 90 °C for 2 h. Two grams of IC (type V) was dissolved in 100 mL of deionized H_2_O at 80 °C for 1 h. PVA: IC (95:5) wt.% was blended. Then, the polymer blend was crosslinked, using 5 g of glutaraldehyde (GA) (Alfa Aesar; 50 wt.% in H_2_O) as a covalent crosslinker, and 5 g of 4-sulfophthalic acid (SPA) (Sigma-Aldrich; 99.9 wt.% in H_2_O) as an ionic crosslinker and sulfonating agent for PVA [24,26]. Then, the inorganic–organic nanocomposite was prepared by incorporating 1, 2.5, 5, and 7.5 wt.% of SO_4_ZrO_2_ into the polymeric matrix. Figure S1, in Supplementary Materials, explains the S-PVA/IC/SO_4_ZrO_2_ membrane structure, within which PVA and IC are ionically crosslinked through the esterification reaction between the carboxylic groups of the sulfophthalic acid and the hydroxyl groups of the polymers. In addition, the acetal reactions between the hydroxyl groups of the polymers and the aldehyde groups of the glutaraldehyde led to the covalent crosslinking of the two polymers. Furthermore, there was the formation of hydrogen bonds between the oxygen-containing SO_4_ZrO_2_ groups and the –OH groups of the PVA and IC [33], respectively.

### 2.2. Characterization

For different samples, after being dried in airflow at room temperature, different tools were used to investigate their properties; a Fourier transform infrared spectrophotometer was used to record the FT-IR spectra (Shimadzu FTIR-8400 S, Shimadzu, Kyoto, Japan), while an X-ray diffractometer was used to evaluate the structures (Shimadzu 7000, Shimadzu, Kyoto, Japan). To trace the thermal characterization of the S-PVA/IC/SO_4_ZrO_2_ membranes, a thermo-gravimetric analyzer (Shimadzu TGA-50, Japan) and a differential scanning calorimeter (DSC) (Shimadzu DSC-60, Japan) were used, and the measurements were carried out under a nitrogen flow of 40 cm^3^∙min^−1^ over a temperature range between room temperature and 800 °C. A scanning electron microscope (SEM) (Joel Jsm 6360LA-Japan) displayed the morphology of the membrane surface, while transmission electron microscopy (TEM, JEM 2100 electron microscope) was used to display the morphology of SO_4_ZrO_2_ nanoparticles. X-ray photoelectron spectroscopy (XPS), with a Phi 5300 ESCA system (Perkin-Elmer, Waltham, WA, USA), was used to investigate the elemental composition of SO_4_ZrO_2_ nanoparticles.

To evaluate the hydrophilicity of the membrane, there was measurement of the contact angle between the membrane surface and the water droplet, using a contact-angle analyzer (Rame-Hart Instrument Co.: Succasunna, NJ, USA, model 500-FI).

The measurement and calculation of the composite membranes’ swelling ratio (SR) and water uptake (WU) are, respectively, shown in Equations (1) and (2), after the sample (1 cm × 1 cm) was left in water overnight, then dried, using tissue to wipe away the surface moisture, and then rapidly weighed [47,48].

(1) $$\mathrm{SR}\left(\%\right)=\frac{L_{\mathrm{wet}}-{\;L}_{\mathrm{dry}}}{L_{\mathrm{dry}}}\times 100$$

(2) $$\mathrm{WU}\left(\%\right)=\frac{W_{\mathrm{wet}\;}-{\;W}_{\mathrm{dry}}}{W_{\mathrm{dry}}}\times 100$$

where *L*_dry_ and *L*_wet_ represent the length of dry and wet composite membranes, while *W*_dry_ and *W*_wet_ are the weight of dry and wet composite membranes, respectively.

Acid-base titration was used to determine the ion exchange capacity (IEC). The weighted samples were placed in 50 cm^3^ of a 2 M NaCl solution for two days, after which titration was completed with a 0.01 N NaOH solution. IEC was calculated as follows [29]:

(3) $$\mathrm{IEC}(\mathrm{meq}/g)=\frac{V_{\mathrm{NaOH}}\times {\;C}_{\mathrm{NaOH}}}{W_{d}}\times 100$$

where C_NaOH_, V_NaOH_, and W_d_ are sodium hydroxide solution concentration, the amount of sodium hydroxide used in titration, and the dry sample weight, respectively.

The dry nanocomposite membranes were put through a tensile strength test at room temperature until they disintegrated. Lloyd Instrument LR10k was used [29].

The membrane of the nanocomposite was placed vertically between two small tanks of 100 mL each in a glass diffusion chamber to test its borohydride permeability. An amount of 1 M NaBH_4_ was poured into the donor tank (A), containing 4 M NaOH solution, which is a common DBFC anolyte. Water was poured inside the receptor tank (B) [29]. Borohydride diffused from A to B across the composite membrane, and the amounts of boron from the ${\mathrm{BH}}_{4}^{-}$ ions transported to tank (B) were measured using an inductively-coupled plasma atomic emission spectrophotometer (ICP-AES, model Prodigy, Teledyne Leeman Labs) every 2 h, four times. Equation (4) [29] was used to determine the borohydride crossover from A to B with time:

(4) $$C_{B}(t)=\frac{A}{V_{B}}\frac{P}{L}C_{A}{(t\;-\;t}_{0})\;$$

where *A* (cm^2^) is the diffusion area, *V*_B_ (cm^3^) is the receptor tank volume, *L* (cm) is the membrane thickness, *C*_B_ and *C*_A_ (mol·L^−1^) are the borohydride concentrations in tanks B and A, respectively, the interval (*t* − *t*_0_) is the time of the ${\mathrm{BH}}_{4}^{-}$ crossover, and *P* is the BH_4_^−^ permeability of the membrane (cm^2^·s^−1^).

The selective nature of the membranes (the ratio of ionic conductivity to borohydride permeability) was determined because it can provide crucial information about the fuel cell’s performance.

The oxidative stability of the membranes produced was determined using the nanocomposite membranes’ weight loss (1.5 × 1.5 cm^2^) in Fenton’s reagent (3 wt.% H_2_O_2_ consisting of 2 ppm FeSO_4_) at 68 °C for 24 h [28].

The electrochemical impedance spectroscopy (EIS) approach was utilized to assess the ionic conductivity of the nanocomposite membranes. A PAR 273A potentiostat (Princeton Applied Research, Inc.: Oak Ridge, TN, USA), connected to a SI 1255 HF frequency response analyzer (FRA, Schlumberger Solartron), was used for this analysis. The membranes were dipped in a 4 M NaOH solution at room temperature for 30 min. [27,29,31]. They were then kept between two stainless steel electrodes at an open circuit potential of 5 mV, with a signal amplitude in the range of 100 Hz to 100 kHz. The ability of the membranes to conduct ions was determined using Equation (5) [28]:

(5) $$\sigma =\frac{d}{RA}$$

where *σ* (S·cm^−1^) is the ionic conductivity of membrane, *R* (Ω) is the membrane resistance, *A* (cm^2^) is the membrane area, and *d* (cm) is the membrane thickness.

The composite membranes were kept in a 0.5 M NaCl solution for one day, and then pre-activated in 2 M NaOH for 4 h to assess their DBFC performance [29]. A membrane with an active area of 50 cm^2^ was used to separate the sides of two fuel cells, vertically, and the performance experiment was conducted in potentiostatic mode at room temperature. For the sake of comparison, Nafion 117 was utilized as a commercial reference membrane.

## 3. Results and Discussion

### 3.1. Characterization of SO_4_ZrO_2_ and Nanocomposite Membranes

**Figure 1:**
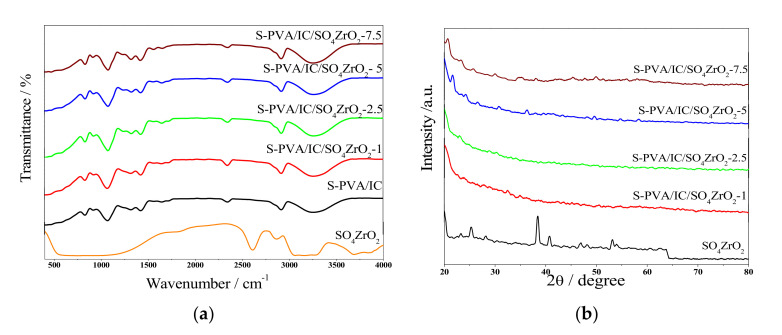
(**a**) FTIR spectra and (**b**) XRD patterns of SO_4_ZrO_2_ and S-PVA/IC/SO_4_ZrO_2_ membranes.

Figure 1a shows the FTIR spectra of SO_4_ZrO_2_. A wide peak of about 3400 cm^−1^ was observed, in addition to the peak at around 1630 cm^−1^. This could be due to the adsorbed H_2_O molecules, as well as the peak around 500 cm^−1^, referred to as the Zr–O band. In the region of 1200–900 cm^−1^, the ${\mathrm{SO}}_{4}^{2-}$ group IR bands were observed [49] with peaks at 1217, 1128, and 1016 cm^−1^, which are characteristic of S–O. The bands at 950 and 1100 cm^−1^ can be attributed to the sulfate groups of doping agents. For the membranes, however, the bands around 3250 cm^−1^ are due to the hydroxyl groups, where H-bonding has a large influence on these bands in PVA and IC. The bands at 1600 cm^−1^ refer to the O–H bonds from water molecules. Their adsorption increases with increasing amounts of sulfated zirconia, due to its hydrophilic properties. The bands at about 2840 and 2300 cm^−1^ were due to the C–H bonds in the structure of the polymers [28], and the characteristic peak for iota carrageenan sulfate groups was seen at 830 cm^−1^. The weak bands at 1700 and 1750 cm^−1^ corresponded to C=O bonds and C–H bonding in the aromatic structure of sulfophthalic acid (SPA), respectively. This demonstrates that the crosslinking process has been completed. As shown in Figure 1b, the amorphous structure of the synthesized membranes grew as the dopant concentration rose, indicating sufficient membrane capacity for excellent ion conduction [50]. However, the sulfated-zirconia-powder curve exhibited typical peaks intensity of SO_4_ZrO_2_ at a 2θ of 28,38,54. [38,51].

**Figure 2:**
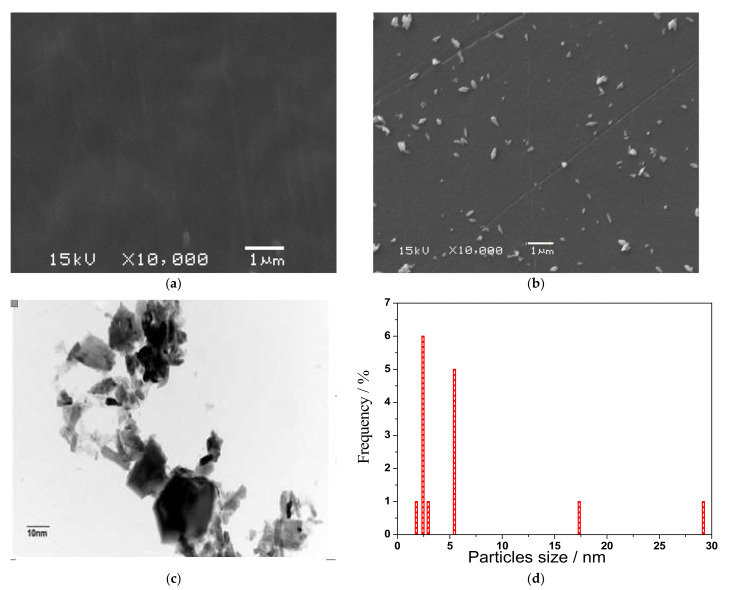
SEM images for (**a**) the undoped membrane surface and (**b**) the S-PVA/IC/SO_4_ZrO_2_-7.5 membrane surface; (**c**) TEM image for SO_4_ZrO_2_nanoparticles and (**d**) frequency distribution plot of SO_4_ZrO_2_ nanoparticles size.

**Figure 3:**
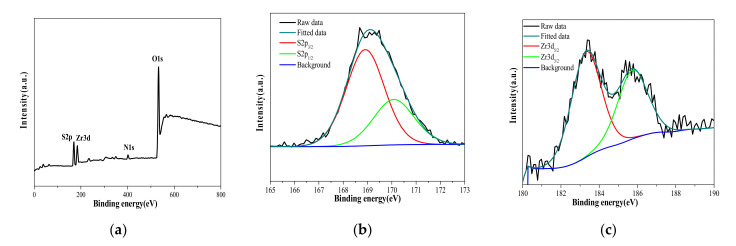
(**a**) XPS survey spectra of SO_4_ZrO_2_, (**b**) S2p spectra, and (**c**) Zr3d spectra of the synthesized SO_4_ZrO_2_ nanoparticles.

Figure 2a,b shows that the surface of the undoped crosslinked membrane had no defects, while for the doped membrane, there was a good dispersion, without agglomeration, of sulfated zirconia. The TEM image in Figure 2c demonstrates that the sulfated zirconia formed nanoscale particles with a small concentration of aggregation. However, XPS elemental analysis was employed to confirm the synthesis of sulfated zirconia, as shown in Figure 3a, where sulfur groups were presented on the surface of SO_4_ZrO_2_. In Figure 3b, the S2p spectrum of the sulfate group can be fitted into two major peaks, at 168.9 and 170.0 eV, assigned to S 2p_3/2_ and S 2p_1/2_ spectra for the oxidized sulfur species, respectively, while the Zr 3d spectrum shown in Figure 3c can be fitted into doublet peaks at approximately 183.4 and 185.8 eV, corresponding to contributions from Zr 3d_5/2_ and Zr 3d_3/2_, respectively.

### 3.2. Thermal and Mechanical Analysis

**Figure 4:**
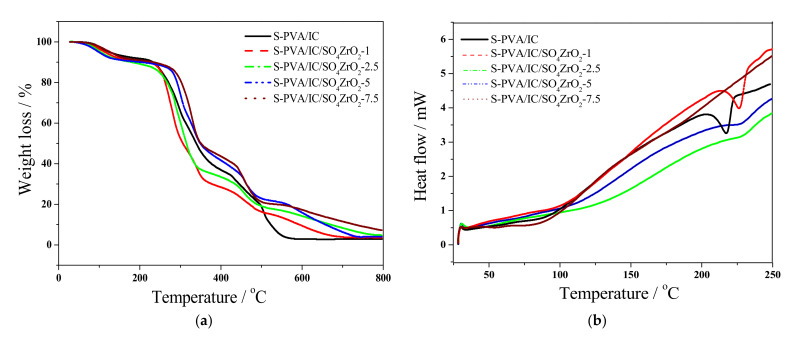
(**a**) TGA and (**b**) DSC curves of nanocomposite membranes.

Figure 4a depicts the TGA curves of polymeric blend membranes without and with SO_4_ZrO_2_. The initial weight loss at approximately 150 °C (8%) is because the moisture evaporated in all membranes [23,52]. The second weight loss of composite membranes happened between 150 and 270 °C, due to the breakdown of functional groups [53,54]. The third weight-loss stage is characterized by a noteworthy breakdown from 270 to 360 °C. This could be attributed to the polymeric chain decomposition [52,55], which began at 230 °C for the undoped membranes, and at 270 °C, with a lower weight percentage, for doped membranes. This behavior demonstrates that the addition of a dopant compound to a composite membrane improves its thermal stability by boosting its covalent, ionic, and hydrogen bonding. Only one endothermic peak in DSC (Figure 3b) indicates complete miscibility of SO_4_ZrO_2_ in the membrane structure, and the absence of this peak at SO_4_ZrO_2_, with a weight percent of 7.5, could be because many hydrogen bonds were formed between the dopant and the polymer structure. This partially destroyed its crystallinity [32,33], leading to a decrease in the membranes’ melting temperature.

**Figure 5:**
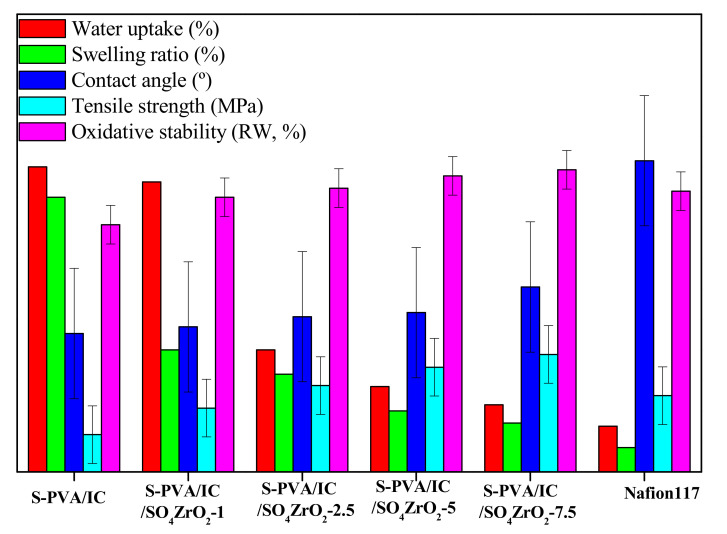
Physicochemical properties of the fabricated membranes and Nafion 117.

The addition of SO_4_ZrO_2_ to the polymeric matrix enhances its mechanical tensile strength [51]. As shown in Figure 5, increasing the incorporation of SO_4_ZrO_2_ into the polymeric matrix increased the tensile strength of the nanocomposite membranes. This is due to the increase in the compatibility of the composite membrane, by increasing the connection between functional groups, such as ${\mathrm{SO}}_{4}^{-}$ and OH groups, as well as the characteristic groups of SO_4_ZrO_2_, of the two polymers, via the formation of hydrogen, ionic, and covalent bonds. These bonds improved the nanocomposite membranes’ interfacial adhesion in comparison to the membranes that were not doped.

Membrane surfaces are hydrophobic when contact angles are below 90°, and hydrophilic when they are more than 90°. The number of water molecules responsible for proton transport in the membrane must be measured; however, the membrane’s ability to absorb more water promotes swelling, which reduces the membrane’s mechanical strength. On the other hand, a lack of water molecules reduces the membrane’s conductivity. This means the conductivity and strength of the membrane have an impact on fuel cell performance. Figure 5 shows the character of the manufactured composite membranes placed in deionized water. It shows that, as the composition of the dopant increased from 1 to 7.5 wt.%, the composite membranes became less hydrophilic and very thick [31,45]. In addition, the swelling ratio and water uptake of the polymeric membranes were reduced, which is highly important [56]. In other words, in comparison to the undoped membrane, an increase in the dopant in the membrane matrix would increase the structure compactness. This prevents excess water in the pathways of the polymeric matrix [57,58,59,60].

### 3.3. Oxidative Stability

Fenton’s reagent test was utilized to determine the chemical stability of the composite membranes. As shown in Figure 5, the chemical stability of the undoped membrane was the lowest, but when SO_4_ZrO_2_ was added as a doping agent, the membranes’ resistance to OOH and OH radical attack was improved. The most stable synthesized membrane was S-PVA/IC/SO_4_ZrO_2_-7.5. It maintained its weight at about 99%, demonstrating that adding SO_4_ZrO_2_ to the polymeric matrix increases its chemical stability [42].

### 3.4. Ionic Conductivity, IEC, and Borohydride Crossover

**Figure 6:**
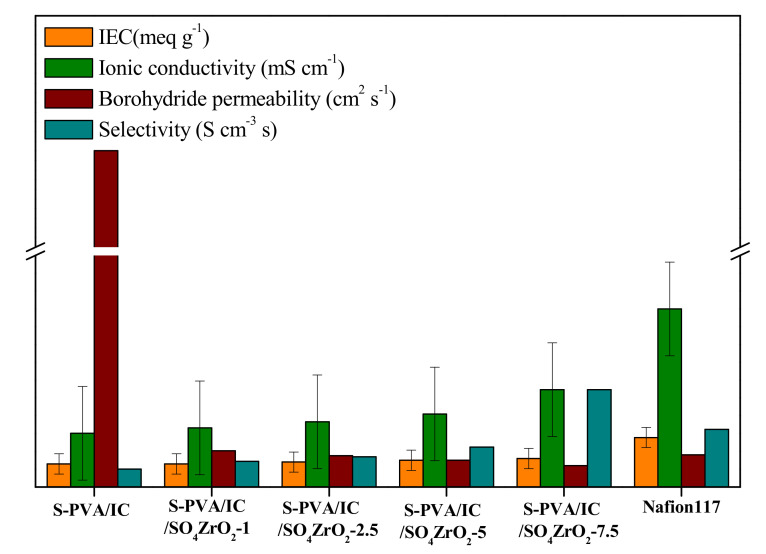
Ion exchange capacity, ionic conductivity, borohydride permeability, and selectivity of the fabricated membranes and Nafion 117.

The ion exchange capacities of the doped and undoped membranes were compared to Nafion 117, as shown in Figure 6. The IEC values, represented in Table S2 in Supplementary Materials, increased as the volume of SO_4_ZrO_2_ in the composite membranes increased. This is because the composite membranes have more acidic exchangeable groups, which increases the charge in the polymeric matrix. Accordingly, the ionic conduction should be improved [45]. For instance, the S-PVA/IC/SO_4_ZrO_2_-7.5 membrane had higher ionic conductivity (21.6 mS·cm^−1^) than the undoped membrane (8.1 mS·cm^−1^), as shown in Figure S2 in Supplementary Materials.

For the fuel permeability of the composite membranes, as shown in Figure 6, it is clear that, by adding SO_4_ZrO_2_ to the polymeric matrix, the fuel permeability decreased. This is because the dopant can constrict the polymeric matrix channels and reduce water uptake, thus decreasing the ${\mathrm{BH}}_{4}^{-}$ permeability [29,31]. However, the higher selectivity of the S-PVA/IC/SO_4_ZrO_2_-7.5 membrane, which is 1.14 × 10^5^ mS·cm^−3^, as compared to the undoped S-PVA/IC membrane, which has a selectivity of around 0.25 × 10^5^ mS·cm^−3^, confirmed that the nanocomposite membranes produced are suitable for use in DBFCs [58].

### 3.5. Fuel Cell Performance

**Figure 7:**
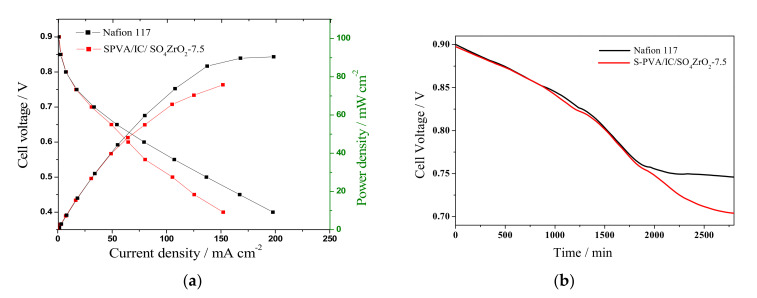
(**a**) Power density curves and polarization of DBFCs, and (**b**) fuel cell stability recorded at a current density of 50 mA·cm^−2^ for 48 h, at room temperature, using PVA/IC/SO_4_ZrO_2_-7.5 and Nafion117 membranes.

As shown in Figure 7a, the performance of the nanocomposite membrane S-PVA/IC/SO_4_ZrO_2_-7.5, which had the highest conductivity and lowest permeability, was tested in a lab DBFC, and compared to that of the Nafion 117^®^ membrane using similar tests. The polarization curves reveal that the S-PVA/IC/SO_4_ZrO_2_-7.5 membrane resulted in lower DBFC discharge currents than Nafion117, because Nafion117 has a higher charge density. In addition, this could be due to electrochemical reactions at the anode and cathode that were restricted by Na^+^ ions’ mass transfer via the S-PVA/IC/SO_4_ZrO_2_-7.5 membrane [29,31]. Furthermore, for an inside view of S-PVA/IC/SO_4_ZrO_2_-7.5 membrane stability, the same tested membranes were also subjected to a 48 h stability test, as shown in Figure 7b. The behavior of both DBFCs was similar for the first 30 h, but after that, the cell voltage of the DBFC with the Nafion117 membrane remained nearly stable, while the cell voltage of the DBFC with S-PVA/IC/SO_4_ZrO_2_-7.5 was reduced. This could be attributed to ohmic losses caused by the increase in the composite membranes’ resistance.

## 4. Conclusions

Low-cost nanocomposite membranes were created using a simple blending and solution-casting method with environmentally friendly polymers. The incorporation of SO_4_ZrO_2_ as a dopant into the polymeric blend appears to improve the characteristics of the membrane, such as its mechanical stability, ionic conductivity, and oxidative stability, as well as its ability to reduce excess water and prevent ${\mathrm{BH}}_{4}^{-}$ crossover, particularly in the nanocomposite membrane with 7.5wt.% SO_4_ZrO_2_. However, under the same experimental conditions, the S-PVA/IC/SO_4_ZrO_2_-7.5 membrane had a slightly lower peak power density, of about 76 mW·cm^−2^, than Nafion 117, which is 91 mW·cm^−2^. In addition, the S-PVA/IC/SO_4_ZrO_2_-7.5 membrane had good membrane stability in DBFCs, and could outperform Nafion 117 in terms of oxidative stability, tensile strength, and ${\mathrm{BH}}_{4}^{-}$ permeability. Moreover, it can commercially compete with Nafion117 due to its eco-friendly raw materials, low cost, and simple, green synthesis with no toxic solvents involved; it can also be easily manufactured industrially. For all previous reasons, the synthesized S-PVA/IC/SO_4_ZrO_2_-7.5 membrane can be utilized as PEM, after some modifications in its structure, to compete with Nafion 117 in all properties.

## Data Availability

Not applicable.

## Supplementary Materials

The following are available online at https://www.mdpi.com/article/10.3390/polym13234205/s1. Figure S1: Possible structure of the S-PVA/ IC/ SO4ZrO2 membrane. Figure S2: Nyquist plot of S-PVA/IC and S-PVA/IC/SO4ZrO2 nanocomposite membranes. Table S1: Physicochemical properties of the fabricated membranes and Nafion 117. Table S2: Ionic conductivity, Borohydride permeability, IEC and selectivity of the fabricated membranes and Nafion 117.
