# Supplementary material for: Design of Promising Green Cation-Exchange-Membranes-Based Sulfonated PVA and Doped with Nano Sulfated Zirconia for Direct Borohydride Fuel Cells
Source: Polymers (Basel). 2021 Nov 30;13(23):4205. doi: 10.3390/polym13234205 (PMC8659521; doi:10.3390/polym13234205)
Supplement: Supplementary file 1 [file polymers-13-04205-s001.zip › polymers-1466252-supplementary.pdf]

Supplementary information

Design of Promising Green Cation Exchange Membranes Based Sulfonated PVA and Doped with Nano Sulfated Zirconia for Direct Borohydride Fuel Cells

Marwa H. Gouda[1], Noha A. Elessawy[2], Sami A. Al-Hussain[3], Arafat Toghan[3,4*]

[1]*Polymer Materials Research Department, Advanced Technology and New Materials Research Institute (ATNMRI), City of Scientific Research and Technological Applications City (SRTA-City), 21934 Alexandria, Egypt*
[2]*Computer Based Engineering Applications Department, Informatics Research Institute IRI, City of Scientific Research and Technological Applications City (SRTA-City), 21934 Alexandria, Egypt*
[3]*Chemistry Department, College of Science, Imam Mohammad Ibn Saud Islamic University (IMSIU), Riyadh 11623, Saudi Arabia*
[4]*Chemistry Department, Faculty of Science, South Valley University, Qena 83523, Egypt*

*Corresponding author E-mail address: (Arafat Toghan): arafat.toghan@yahoo.com; aatahmed@imamu.edu.sa

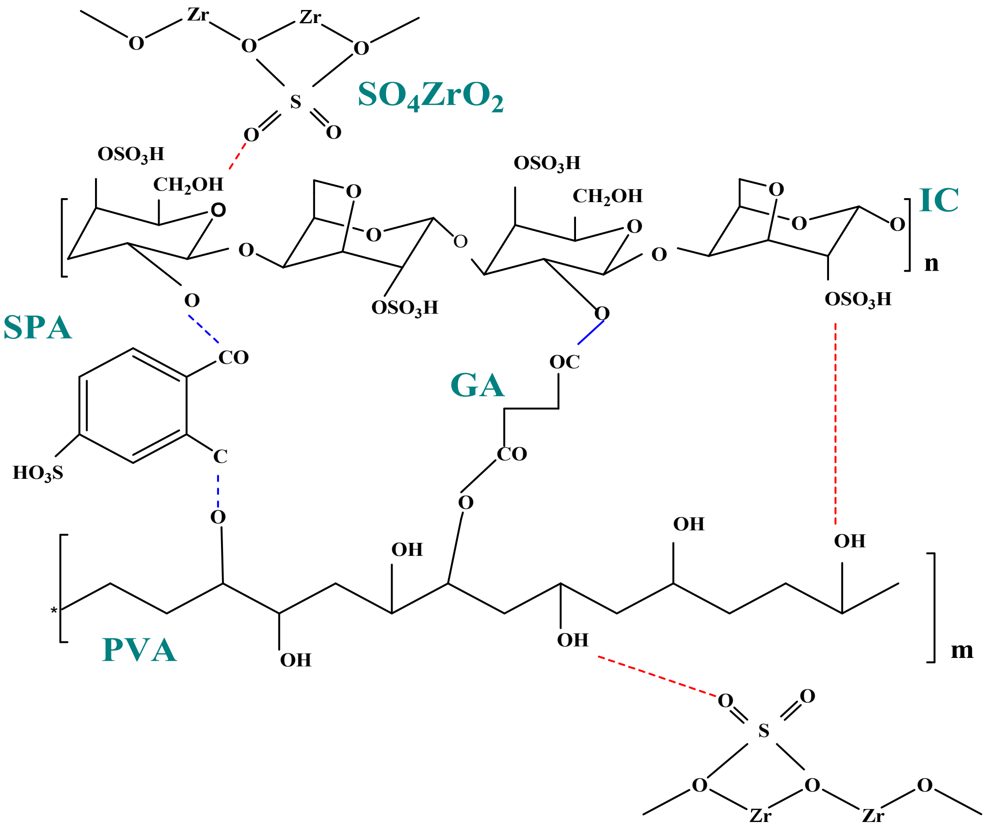

Figure S1: Possible structure of the S-PVA/ IC/ SO_4ZrO_2 membrane

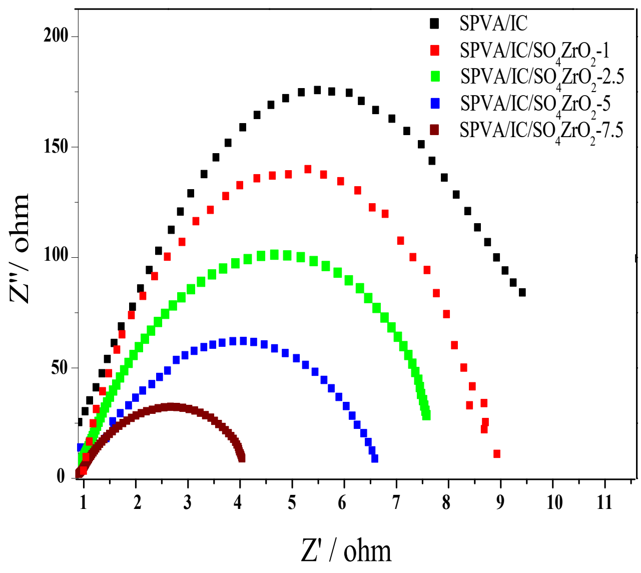

Figure S2: Nyquist plot of S-PVA/IC and S-PVA/IC/SO4ZrO$_2$ nanocomposite membranes.

Table S1: Physicochemical properties of the fabricated membranes and Nafion 117.

Membrane	Thickness (µm)	WU (%)**	SR (%)**	Contact angle (°)**	Tensile strength (MPa)**	Oxidative stability (RW, %)*,**
S-PVA/IC	110	> 100	90±0.3	45.36±0.1	12.2±0.4	81±0.63
S-PVA/IC/ SO_4ZrO_2-1	154	95±0.5	40±0.2	47.53±0.1	20.9±0.4	90±0.63
S-PVA/IC/ SO_4ZrO_2- 2.5	169	40±0.2	32±0.1	50.86±0.1	28.3±0.4	93±0.63
S-PVA/IC/ SO_4ZrO_2- 5	173	28±0.1	20±0.1	52.21±0.1	34.3±0.4	97±0.63
S-PVA/IC/ SO_4ZrO_2-7.5	179	22±0.08	16±0.1	60.60±0.1	38.5±0.4	99±0.63
Nafion 117	170	9.5	13	102	25	92

*The retained weight of membranes (RW) after immersion for a day in Fenton's reagent.

**The measurements were replicated three times for the same prepared membranes and the standard deviation was evaluated accordingly for all tests.

Table S2: Ionic conductivity, Borohydride permeability, IEC and selectivity of the fabricated membranes and Nafion 117.

Membrane	IEC (meq g^{-1})*	Ionic conductivity (mS cm^{-1})*	Borohydride permeability ($10^{-6} cm^2 s^{-1}$)	Selectivity (10^5 S cm^{-3} s)
S-PVA/IC	0.12±0.28	8.1±0.13	3.8	0.021
S-PVA/IC/ SO_4ZrO_2-1	0.13±0.28	10.6±0.13	0.31	0.34
S-PVA/IC/ SO_4ZrO_2- 2.5	0.15±0.28	12.9±0.13	0.29	0.44
S-PVA/IC/ SO_4ZrO_2- 5	0.21±0.28	14.8±0.13	0.23	0.64
S-PVA/IC/ SO_4ZrO_2-7.5	0.24±0.28	21.6±0.13	0.10	2.16
Nafion 117	0.89	45.0	0.40	1.12

*The measurements were replicated three times for the same prepared membranes and the standard deviation was evaluated accordingly for all tests.
